# Selective sinoatrial suppression during post-ablation adenosine testing without atrioventricular block

**DOI:** 10.1093/ehjcr/ytag002

**Published:** 2026-01-13

**Authors:** Sudipta Mondal, Nadeem Afroz Muslim

**Affiliations:** Department of Cardiology, The Mission Hospital, Immon Kalyan Sarani, Sector IIC, Bidhannagar, Durgapur, West Bengal, PIN 713212, India; Department of Cardiology, The Mission Hospital, Immon Kalyan Sarani, Sector IIC, Bidhannagar, Durgapur, West Bengal, PIN 713212, India

## Case

**Figure 1:**
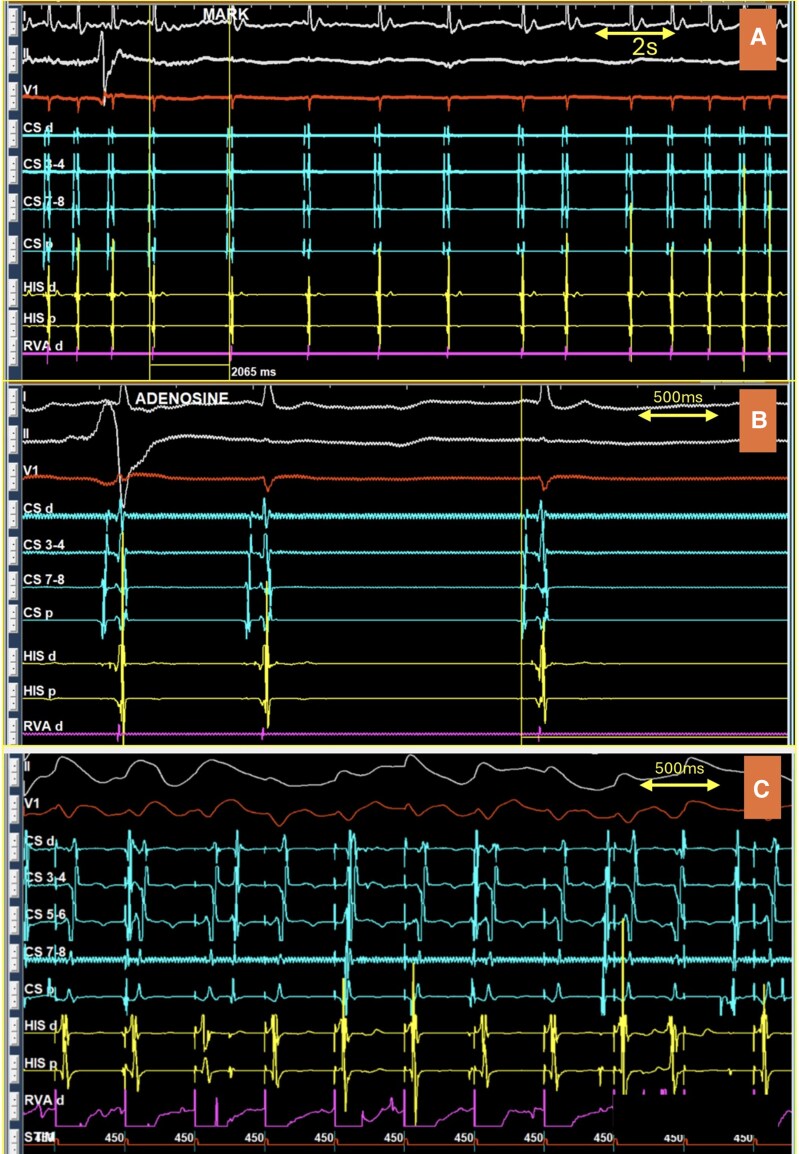
*(A)* Post-ablation adenosine challenge (12 mg IV push) caused sinus node dysfunction without affecting atrioventricular nodal conductivity (Not shown: Post-ablation study revealed a baseline Atriohisian (AH) interval of 65 ms and a His-ventricle (HV) interval of 40 ms at a sinus cycle length of 770 ms. Incremental atrial pacing demonstrated decremental nodal conduction, evidenced by a progressive AH interval prolongation, leading to atrioventricular Wenckebach periodicity at a cycle length of 290 ms. Atrial extrastimulus testing corroborated these findings, showing similar decremental behaviour with AH block occurring at S1: S2 of 450:230 ms. The AH interval in the last conducted beat of this sequence measured 159 ms. Dual AV nodal physiology was ruled out); *(B)* Enlarged view showing sinus slowing without significant change in AH or HV intervals; *(C)* Ventriculoatrial dissociation during ventricular burst pacing showing no evidence of retrograde accessory pathway conduction.

A 45-year-old male presented with symptomatic orthodromic atrioventricular (AV) receprocating tachycardia (AVRT) mediated by a left lateral accessory pathway (AP). He underwent successful radiofrequency (RF) ablation, with acute success confirmed by the loss of pre-excitation and retrograde AP conduction. To definitively exclude residual AP conduction, a post-ablation adenosine challenge (12 mg IV push) was performed; unexpectedly, instead of the typical brief AV block (*Figure 1A,B*), the patient developed sinus node dysfunction (SND) manifesting as a period of sinus slowing. To rule out inadequate intravenous delivery as the cause of the initial response, an 18 mg dose of adenosine was administered again. This higher dose reproduced the same isolated sinus node suppression without AV block. The baseline sinus node function was normal. Post-ablation decremental AV conduction, a slight but insignificant prolongation of the atriohisian (AH) interval (65 to 75 ms) following adenosine administration, and the presence of ventriculoatrial dissociation (*Figure 1C*) collectively rendered an AH AP an unlikely diagnosis.

Adenosine exerts its negative dromotropic effects primarily through the activation of the A1 receptor (A_1_R), leading to a direct (cAMP-independent) activation of the inwardly rectifying potassium currents, specifically IK _Ado, Ach_.^1–3^ A_1_R heterogeneity (relative abundance of A_1_R in different cardiac structures; internodal and intranodal heterogeneity) may represent the sole cause for varying sensitivities to intravenous adenosine. This observation may partially account for the occasional therapeutic failure of adenosine in managing AV node-dependent supraventricular tachycardias. However, detailed studies on the comparative A_1_R density between the sinoatrial (SA) and AV nodes remain limited.

In summary, this case highlights rare, selective SA node sensitivity to adenosine that spares the AV node, which potentially complicates the accurate assessment of ablation success via adenosine challenge. Given the probable transient nature of this phenomenon, the requirement for pacing or atropine is considered low, and a repeat adenosine challenge is likely redundant.

## Data Availability

No new data were generated or analysed in support of this research.

## Notes

**Consent:** Obtained from the patient in line with COPE guidance.
